# Comparative Analysis of Healthcare Quality Between the University Hospital of the West Indies and Public Hospitals in Jamaica

**DOI:** 10.7759/cureus.73423

**Published:** 2024-11-11

**Authors:** Cornelius Neptune, Alicia Edwards, Marson Taylor, Britony Dixon

**Affiliations:** 1 Community Health, University of the West Indies Mona, Kingston, JAM; 2 Community and Family Medicine, University of the West Indies Mona, Kingston, JAM

**Keywords:** healthcare quality, jamaica, patient experience, patient satisfaction, public hospitals, university hospital of the west indies (uhwi)

## Abstract

Objective

This study aims to assess the quality of healthcare services at the University Hospital of the West Indies (UHWI), a quasi-public hospital, compared to public hospitals in Jamaica, examining both patient and clinician perceptions.

Methods

This cross-sectional study included one hundred patients (30 males, 70 females) aged 22 to 95, and 52 clinicians (29 females, 23 males) from 10 hospitals, comprising UHWI and nine public hospitals, were surveyed. Quality assessment encompassed structural adequacy, time management, privacy protection, competency, and healthcare standards. Data collection occurred through both interview-administered and self-administered questionnaires. Statistical analysis was then done using the chi-square test.

Results

Clinicians at UHWI perceived structural adequacy differently than public hospitals, with 63% (n=10) agreeing on cubicle space sufficiency at UHWI versus disagreement among public hospital clinicians. Consultation times were varied, with longer durations reported at UHWI. Privacy concerns were more pronounced among UHWI patients. Overall, patient satisfaction was higher at public hospitals (n=56.80%) compared to UHWI (n=15.50%).

Conclusion

The study compared healthcare quality and patient satisfaction between quasi-public and public hospitals in Jamaica. Contrary to expectations, healthcare quality was the same between the two types of hospitals. Patients at public hospitals reported higher satisfaction levels, challenging the assumption that paying for services correlates with higher satisfaction. The study underscores the complexity of assessing healthcare quality. It suggests that investments in resources and service delivery are needed across all hospitals to improve patient experiences and outcomes in Jamaica.

## Introduction

Previous research suggests a disparity in satisfaction levels between private and public hospitals, with the former often outperforming the latter [1]. This discrepancy is often attributed to the perceived superior quality of care private healthcare professionals provide. Other factors include timeliness and superior resources [2]. However, healthcare quality assessment encompasses various dimensions, including structural adequacy and service delivery. While private clinics are often better equipped, public healthcare systems may need help with timeliness and hospitality in service delivery [3,4].

In Jamaica, where public healthcare facilities are more commonly utilized due to cost considerations, the government's removal of user fees at public hospitals in 2008 aimed to promote universal access to healthcare [5]. However, certain institutions, such as the University Hospital of the West Indies (UHWI), retained user fees, leading to concerns about potential impacts on healthcare quality [6,7].

Despite these concerns, no studies have directly compared the quality of care between user-fee hospitals like UHWI and non-user-fee public hospitals in Jamaica. Therefore, the objective of this study is to assess whether the quality of healthcare provided at UHWI differs from that offered at public hospitals based on perceptions from both patients and clinicians. By shedding light on the current state of quasi-public and public healthcare systems, this study aims to inform policymakers, such as the Ministry of Health, on potential measures to enhance healthcare services and delivery in Jamaica.

## Materials and methods

A cross-sectional research method was implemented for this study. Given the study's focus on assessing healthcare in quasi-public and public hospitals, the population included clinicians and patients across Jamaica. Participating hospitals encompassed UHWI, Kingston Public Hospital, Noel Holmes Hospital, Cornwall Regional Hospital, Mandeville Regional Hospital, Falmouth Hospital, Savanna La Mar Public General Hospital, St Ann's Hospital, Spanish Town Hospital, and Port Maria Hospital.

Inclusion criteria included all patients diagnosed with diabetes, hypertension, or both who attended the clinic at that hospital for that condition, aged 18 and over. All clinicians, ranging from interns to residents, employed at the specific institutions treated diabetic or hypertensive patients in their clinics. Exclusion criteria included patients without diabetes or hypertension, patients who did not attend clinics for the treatment of diabetes or hypertension, patients attending these clinics for the first time, patients under 18 years old, and consultant physicians.

The sample comprised 100 patients and 52 clinicians. Thirty percent of patients and clinicians were from UHWI, and 70% were from public hospitals. After quantitative data collection, an additional sample of clinicians was selected using purposive sampling to ensure respondents had prior experience treating patients diagnosed with diabetes, hypertension, or both. A total of 52 clinicians were then selected.

The study employed a questionnaire and an interview guide for data collection, utilizing a combined qualitative and quantitative approach. Quality assessment was based on structure, time, privacy, competence, and the standard of healthcare provided. Two questionnaires were developed for clinicians and patients, respectively. The patient questionnaire, comprising 18 questions, covered demographics, services provided at the hospital, and the delivery of those services. The clinician questionnaire encompassed demographics, healthcare structure, delivery, and standards, totaling 16 questions.

A pilot study was conducted to assess the suitability and applicability of the scales used. Five clinicians and 10 patients were conveniently selected from UHWI for the pilot study. Participants could interpret or answer the questions quickly, leading to revisions and modifications to the questionnaires as necessary.

The study's aims and objectives were explained to patients and clinicians, and verbal consent was obtained along with written consent forms. Confidentiality was assured, and the researcher personally administered questionnaires. Additional explanations were provided to participants who had difficulty understanding the questions.

Quantitative data were analyzed using SPSS Statistics version 22 (IBM Corp. Released 2013. IBM SPSS Statistics for Windows, Version 22.0. Armonk, NY: IBM Corp.), with descriptive statistics computed for continuous and categorical variables. Differences were tested using chi-square and t-tests, with statistical significance at p<0.05.

## Results

The perspectives of clinicians regarding the resources and amenities available at different healthcare institutions reveal significant disparities in various aspects (Table 1). For instance, 62.5% (n=10) of clinicians at UHWI acknowledged the space sufficiency of cubicles, while 53% (n=19) of clinicians at public hospitals disagreed. Many clinicians at UHWI (n=11.69%) and public hospitals (n=17.47%) agreed that the cubicles offer adequate privacy. Similarly, most clinicians at UHWI (n=10.63%) and public hospitals (19.53%) reported the availability of essential medical equipment. However, a notable discrepancy emerged regarding the adequacy of medical supplies, with a heavily neutral and in agreement stance at UHWI (n=10.63%), compared to 56% (n=20) disagreeing at public hospitals. Clinicians at UHWI again expressed a neutral stance regarding staffing adequacy, whereas 67% (n=24) of public hospital clinicians felt insufficient staffing.

**Table 1:** Comparison of resources and amenities provided at UHWI and public hospitals in Jamaica (clinicians) Data has been represented as % (n). N represents the total population. p<0.05 UHWI: University Hospital of the West Indies

Resources and amenities	Agreement level	UHWI (N=16)	Public hospital (N=36)	P-value
Basic medical equipment	Strongly agree	0.00 (0)	8.33 (3)	0.49
	Agree	62.50 (10)	44.44 (16)	
	Neutral	18.75 (3)	19.44 (7)	
	Disagree	12.50 (2)	25.00 (9)	
	Strongly disagree	6.25 (1)	2.78 (1)	
Sufficient cubicles	Strongly agree	0.00 (0)	8.33 (3)	0.07
	Agree	62.50 (10)	22.22 (8)	
	Neutral	25.00 (4)	16.67 (6)	
	Disagree	6.25 (1)	33.33 (12)	
	Strongly disagree	6.25 (1)	19.44 (7)	
Privacy	Strongly agree	0.00 (0)	5.56 (2)	0.136
	Agree	68.75 (11)	41.67 (15)	
	Neutral	12.50 (2)	25.00 (9)	
	Disagree	12.50 (2)	22.22 (8)	
	Strongly disagree	6.25 (1)	5.56 (2)	
Sufficient supplies	Strongly agree	0.00 (0)	0.00 (0)	0.182
	Agree	37.50 (6)	19.44 (7)	
	Neutral	25.00 (4)	25.00 (9)	
	Disagree	31.25 (5)	47.22 (17)	
	Strongly disagree	6.25 (1)	8.33 (3)	
Sufficient staffing	Strongly agree	0.00 (0)	0.00 (0)	0.173
	Agree	37.50 (6)	16.67 (6)	
	Neutral	25.00 (4)	16.67 (6)	
	Disagree	31.25 (5)	41.67 (15)	
	Strongly disagree	6.25 (1)	25.00 (9)	

The clinicians' perspectives on hospital service delivery revealed diverse viewpoints on several key aspects, including unnecessary waiting times, consultation durations, patient privacy, and adherence to international guidelines. Notably, a significant proportion of clinicians, 56% (n=9) at UHWI and 61% (n=22) at public hospitals expressed concerns or neutrality regarding unnecessary waiting times (Table 2). Regarding patient privacy and confidentiality, consensus prevailed among clinicians from UHWI and public hospitals.

**Table 2:** Clinician's assessment of patient experience parameters at UHWI and public hospitals in Jamaica Data has been represented as % (n). N represents the total population. p<0.05 UHWI: University Hospital of the West Indies

Parameter	Agreement level	UHWI (N=16)	Public hospital (N=36)	P-value
Unnecessary waiting	Strongly agree	12.50 (2)	2.78 (1)	0.137
	Agree	43.75 (7)	36.11 (13)	
	Neutral	12.50 (2)	41.67 (15)	
	Disagree	31.25 (5)	19.44 (7)	
	Strongly disagree	0.00 (0)	0.00 (0)	
Adequacy of patient privacy	Strongly agree	6.25 (1)	11.11 (4)	0.266
	Agree	56.25 (9)	38.89 (14)	
	Neutral	12.50 (2)	36.11 (13)	
	Disagree	25.00 (4)	13.89 (5)	
	Strongly disagree	0.00 (0)	0.00 (0)	
Adequacy of patient confidentiality	Strongly agree	25.00 (4)	38.89 (14)	0.76
	Agree	62.50 (10)	50.00 (18)	
	Neutral	12.50 (2)	8.33 (3)	
	Disagree	0.00 (0)	0.00 (0)	
	Strongly disagree	0.00 (0)	2.78 (1)	

Regarding consultation durations, most clinicians in both settings typically spent 30 minutes or less with patients (Table 3).

**Table 3:** Service delivery: consultation times as reported by clinicians Data are represented as % (n). N represents the total population. p<0.05 HWI: University Hospital of the West Indies

Consultation time (min)	UHWI (N=16)	Public hospital (N=36)	P-value
<30	50.00 (8)	55.60 (20)	0.32
30-45	31.30 (5)	38.90 (14)	
46-60	18.80 (3)	5.60 (2)	

There were similarities in how frequently clinicians explained procedures to patients and assessed their comprehension. However, there was a slight discrepancy in the performance of physical examinations, with UHWI clinicians consistently conducting them compared to slightly fewer clinicians at public hospitals.

Table 4 shows an assessment of the standard of care. This indicated varying perceptions, with a notable proportion of clinicians at both institutions expressing neutrality. Regarding upholding international standards of practice, 75% (n=12) of clinicians at UHWI felt they were being maintained; in comparison, 56% (n=20) of clinicians at public hospitals believed otherwise. Responses were similar when assessing the clinicians' perception of the quality of care offered.

**Table 4:** Clinician's perception of the standard of care offered Data are represented as % (n). N represents the total population. p<0.05 HWI: University Hospital of the West Indies

Parameter	Agreement level	UHWI (N=16)	Public hospital (N=36)	P-value
International guideline upheld	Strongly agree	18.75 (3)	11.11 (14)	0.33
	Agree	56.25 (9)	33.33 (12)	
	Neutral	12.50 (2)	38.89 (14)	
	Disagree	6.25 (1)	13.89 (5)	
	Strongly disagree	6.25 (1)	2.78 (1)	
Consultation availability	Strongly agree	25.00 (4)	30.56 (11)	0.45
	Agree	56.25 (9)	41.67 (15)	
	Neutral	18.75 (3)	16.67 (6)	
	Disagree	0.00 (0)	8.33 (3)	
	Strongly disagree	0.00 (0)	2.78 (1)	
Good quality care offered	Strongly agree	6.25 (1)	0.00 (0)	0.02
	Agree	68.75 (110	13.89 (5)	
	Neutral	25.00 (4)	63.89 (23)	
	Disagree	0.00 (0)	22.22 (8)	
	Strongly disagree	0.00 (0)	0.00 (0)	

Table 5 highlights patients' views on resources and amenities at the institutions. Similarities were observed across various parameters, except for the availability of clinicians, where more patients felt there was inadequate staffing at the public hospitals. Furthermore, many patients at both institutions found the privacy maintenance adequate.

**Table 5:** Comparison of resources and amenities provided at UHWI and public hospitals in Jamaica (patients) Data are represented as % (n). N represents the total population. p<0.05 UHWI: University Hospital of the West Indies

Resources and amenities	Agreement	UHWI (N=30)	Public (N=70)	P-value
Need to visit private labs for further investigations	Yes	33.33 (10)	41.43 (29)	0.591
	No	66.67 (20)	58.57 (41)	
Seats	Yes	73.33 (22)	72.86 (51)	1
	No	26.67 (8)	27.14 (19)	
Sufficient clinicians	Yes	53.33 (16)	44.29 (31)	0.54
	No	46.67 (14)	55.71 (39)	
Privacy	Yes	80.00 (24)	90.00 (63)	0.299
	No	20.00 (6)	10.00 (7)	

Many patients at UHWI and public hospitals encountered waiting times surpassing 60 minutes. Consultation durations were typically considered adequate (Table 6).

**Table 6:** Service delivery assessment based on the patient's perspective Data are represented in % (n). N represents the total population. p<0.05 HWI: University Hospital of the West Indies

		UHWI (N=30)	Public hospital (N=70)	P-value
Consultation time	<10	0.00 (0)	7.10 (5)	0.019
	10-15	36.70 (11)	52.90 (37)	
	16-30	36.70 (11)	34.3 (24)	
	31-45	20.00 (6)	2.90 (2)	
	46-60	6.70 (2)	2.90 (2)	
Sufficient consultation times	yes	73.30 (22)	87.00 (60)	0.173
	No	26.70 (8)	13.00 (9)	
Waiting times	<30	3.30 (1)	4.30 (3)	0.08
	30-45	23.20 (7)	7.20 (5)	
	45-60	23.30 (7)	15.90 (11)	
	>60	50.00 (15)	72.50 (50)	

Table 7 shows a higher percentage of patients at public hospitals (n=56.80%) expressed extreme satisfaction with the service than those at UHWI (n=15.50%). Although there were numerous positive similarities in patients' perceptions regarding effective communication and clinicians' assessment of patients' knowledge and privacy during consultations, slightly better results were observed among patients from public hospitals.

**Table 7:** Comparison of patient's level of satisfaction at UHWI and public hospitals in Jamaica Data are represented in % (n). N represents the total population. p<0.05 UHWI: University Hospital of the West Indies

Satisfaction level	UHWI (N=30)	Public hospital (N=70)	P-value
Very dissatisfied	6.67 (2)	2.86 (2)	0.027
Dissatisfied	16.67 (5)	7.14 (5)	
Satisfied	26.67 (8)	10.00 (7)	
Very satisfied	50.00 (15)	80.00 (56)	

## Discussion

The findings of this study provide valuable insights into the quality of healthcare delivery in both quasi-public and public hospitals in Jamaica. Contrary to the initial hypothesis positing potential superiority in quasi-public hospitals due to fee-based services, the results indicate no significant difference in perceived quality between the two institutions [1,8].

Evaluating resources and amenities revealed that patients at UHWI and those in public hospitals generally perceived the available resources as satisfactory. This challenges the assumption that paying for services correlates directly with access to superior resources, as patients receiving free public healthcare also expressed satisfaction with the available facilities. However, clinicians at UHWI were more inclined to view specific resources, such as cubicles, as adequate compared to their counterparts in public hospitals. This disparity is unsurprising, as public hospitals were not originally designed to accommodate the continuously increasing patient load. Clinicians often find themselves sharing cubicles intended for a single doctor's use. Therefore, urgent physical infrastructure enhancements are needed to adequately cater to staff and patients.

There was no statistically significant difference in patients' perceptions of resources and amenities between UHWI and public hospitals. This finding may be anticipated among UHWI patients, considering its smaller patient demographic and the supplementary revenue generated from patient fees complements government resources. However, public hospitals rely solely on government funding and grapple with the challenge of serving a considerably larger patient population, thereby straining available resources. Patients accessing public healthcare services may acknowledge these limitations and perceive the resources as adequate within this context rather than comparing them against an ideal or international standard of care. This contrasts with findings from a study by Peabody et al., which found private clinics to be better equipped and supplied than general public clinics.

Statistically significant findings indicate that consultation times at public institutions are typically shorter than those at UHWI, as perceived by patients. This discrepancy is logically attributed to the higher patient volume in public institutions [7]. Interestingly, despite shorter consultation times in the public setting, patients from both groups reported an average visit duration of 10-30 minutes. Similarly, clinicians from both groups had consultations lasting less than 30 minutes, with most patients deeming this duration sufficient.

Moreover, more patients at UHWI expressed concerns about the confidentiality of their information compared to those at public institutions. This concern stems from UHWI's status as a teaching hospital, where patients may anticipate sharing their health information with medical students and other healthcare professionals. It is imperative to inform patients attending these clinics about this possibility and to request their permission when necessary. Additionally, a higher percentage of UHWI patients believed there was an adequate number of clinicians compared to those in public hospitals.

Notably, a statistically significant majority of patients in public hospitals (n=56.80%) reported being extremely satisfied with healthcare quality, compared to only 50% (n=15) of patients at UHWI. This finding contrasts with Taner and Antony's study, which suggested that private care patients experience higher quality due to payment. However, patient satisfaction is influenced by expectations and past experiences. Since UHWI patients pay for services, they may expect a superior experience to those receiving free services at public hospitals. Additionally, patients who have experienced healthcare at both institutions may have compared them when deciding, further influencing their satisfaction levels.

The disparities in clinician perceptions regarding the sufficiency of resources and amenities, such as cubicles, medical equipment, and staffing levels, highlight areas where improvements are needed in public hospitals to ensure equitable access to quality healthcare. Moreover, the differences in patient satisfaction levels between UHWI and public hospitals underscore the importance of factors beyond financial resources, such as effective communication, patient-centered care, and overall service delivery, in shaping patient perceptions of healthcare quality.

Overall, these findings suggest that while both quasi-public and public hospitals in Jamaica offer generally satisfactory healthcare services, there are opportunities for improvement in infrastructure, service delivery, and patient satisfaction in public hospital settings. Further research is warranted to explore the underlying factors contributing to these disparities and to inform targeted interventions to enhance healthcare quality across all healthcare institutions in Jamaica.

Expanding the scope of the study to include data from emergency and inpatient settings would provide a more comprehensive understanding of the healthcare sector. This broader perspective would offer insights into the quality of care across various healthcare contexts and patient populations. Additionally, considering patients seeking care for conditions beyond diabetes and hypertension would enhance the generalizability of the findings, allowing for a more representative assessment of overall healthcare quality.

Incorporating perspectives from a broader range of healthcare professionals, such as nurses and porters, would enrich the study by capturing diverse viewpoints and experiences within the healthcare team. Nurses play a critical role in patient care, and their insights can provide valuable information on service delivery and resource adequacy. Similarly, perspectives from porters, often involved in patient transport and logistics, could shed light on operational challenges and areas for improvement in hospital infrastructure and workflow.

The study's reliance on patient perceptions may also introduce bias, as individual experiences and expectations vary widely. Additionally, time constraints limited the sample size, which may affect the robustness and generalizability of the findings. Equal recruitment was not possible due to the smaller population at UHWI, adding another limitation to the study. Another limitation of this study was the challenge of finding relevant, up-to-date studies to support the discussion, as most of the available references were outdated.

Future research endeavors should address these limitations by incorporating a broader range of perspectives and contexts, allowing for a more extended data collection period, and ensuring more balanced recruitment across different healthcare facilities. This would provide a more nuanced understanding of healthcare quality and delivery.

## Conclusions

These findings suggest that the payment model only partially determines Jamaica's healthcare delivery quality. Both patients and clinicians perceive certain aspects of healthcare delivery similarly across quasi-public and public hospitals. The study underscores the need for continued investment in infrastructure and resources in public hospitals to ensure equitable access to quality healthcare for all patients. Future research should consider expanding the scope to include other healthcare settings and involve a broader range of healthcare professionals to comprehensively assess healthcare quality in Jamaica. Ultimately, these insights can inform policy decisions to improve the country's healthcare delivery and patient outcomes.

## Appendices

**Table 8:** Questionnaire for clinicians at UHWI and public hospitals UHWI: University Hospital of the West Indies

Question	Response				
Gender	Male	Female			
Title	Intern	Senior house pfficer	Medical officer	Resident	
Level of agreement on cubicle sufficiency	Strongly agree	Agree	Neutral	Disagree	Strongly disagree
Level of agreement on cubicle privacy	Strongly agree	Agree	Neutral	Disagree	Strongly disagree
Level of agreement on the availability of basic medical equipment	Strongly agree	Agree	Neutral	Disagree	Strongly disagree
Level of agreement on the sufficiency of medical supplies	Strongly agree	Agree	Neutral	Disagree	Strongly disagree
Level of agreement on adequate staffing	Strongly agree	Agree	Neutral	Disagree	Strongly disagree
Level of agreement on unnecessary waiting times	Strongly agree	Agree	Neutral	Disagree	Strongly disagree
Level of agreement on consultation availability	Strongly agree	Agree	Neutral	Disagree	Strongly disagree
Average consultation times	<30 min	30 – 45 min	46- 60 min	>60 min	
Level of agreement on privacy/confidentiality maintenance	Strongly agree	Agree	Neutral	Disagree	Strongly disagree
Frequency of explanations given to patients	Always	Sometimes	Rarely		
Assesment of patients’ understanding	Always	Sometimes	Rarely		
Performance of examinations on patients	Yes	No			
Level of agreement on adherence to international guidelines	Strongly agree	Agree	Neutral	Disagree	Strongly disagree
Overall adequacy of the quality of care provided	Excellent	Good	Neutral	Adequate	Poor

**Table 9:** Questionnaire for patients at UHWI and public hospitals UHWI: University Hospital of the West Indies

Question	Response				
Gender	Male	Female			
Age	<60	>60			
Chronic illness	Diabetes mellitus	Hypertension	Both		
Highest education level	Primary	Secondary	Tertiary	Other	
Private lab available	Yes	No			
Privacy maintained	Yes	No			
Available seating in the waiting area.	Yes	No			
Sufficient doctor	Yes	No			
Waiting times	<30 min	30-45 min	46-60 min	>60 min	
Reasonable waiting time	<30 min	30-45 min	46-60 min	>60 min	
Average consultation times	<10 min	10-15 min	16-30 min	31-45 min	46-60 min
Consultation time sufficient	Yes	No			
Friendly staff	Yes	No			
Physical examination performed	Yes	No			
Discussed your expectations	Yes	No			
Assessed understanding	Yes	No			
Privacy/confidentiality maintained	Yes	No			
Overall Satisfaction with the service provided	Extremely	Neutral	Slightly	Dissatisfied	
